# Free or Sober?

**Published:** 1893-12-16

**Authors:** 


### The Hospital

An Institutional Journal of

Science, Medicine, Hygiene, Nursing, and News.

*For Hospitals, Asylums, Infirmaries, Dispensaries, Medical Practitioners, Students, and Nurses.*

Vol. XV.—No. 377.] [Dec. 16, 1893.

### Free or Sober?

The late Archbishop of York, the genial and brilliant Dr. Magee, declared on a memorable occasion in the House of Lords, that he "would rather see England free than sober." The British Medical Association, as represented by Dr. Norman Kerr and other officials, has announced to the Home Secretary, and through him to the world, that it would rather see England locked-up than drunk. It has been hinted in high quarters—for example, in the leader columns of the *Times*—that the deputation representing the Association, which pressed "compulsion" upon the Home Secretary last week, was not without a strong element of fanaticism, teetotal fanaticism. Now we shall not deny that there are teetotalers, thousands of them, who are fanatics of the narrowest, least rational, and most blatant type. But medical men, abstainers or otherwise, are seldom of this class, and it is quite certain that the official representatives of the British Medical Association are as far removed from fanaticism as they can well be. There is an explanation of the action of the Association in asking for compulsion which is quite different from this. The plain fact is that doctors have found by experience that habitual drunkards can be cured by one means, and by one means only, the compulsory cutting off of all alcoholic stimulants for an adequate length of time. Inasmuch as the chief business of the doctor is to cure his patient, it is hardly to be wondered at that he is prepared to go all reasonable lengths to effect his laudable object, even to the length of the deprivation of personal liberty. We insist upon this point with some emphasis. The public, very wisely, attaches a great deal of importance to medical opinion on the "drink" question; and it is necessary that the public should thoroughly understand that medical men take up their attitude on the question almost entirely from the point of view of the "cure" and physical well-being of their patients. The merely fanatical, or even moral, or political, hardly enters into their calculations at all.

Few people will experience any difficulty in understanding the doctor's attitude on this question, and all right-thinking persons will be pleased to find that there is such a degree of honest enthusiasm among medical men in the prosecution of their eminently useful and noble art. But when all this is seen and admitted, it still remains true that the question of compulsory detention of habitual drunkards is a political question. It is political, and "therefore it is not a question for doctors to settle," affirm the opponents of compulsion. But why may not doctors settle the question because it is political? In politics, as in other things, knowledge, experience, and capacity are the factors of successful work. Is there any class of persons in the State who have more knowledge of habitual drunkards than doctors, or more experience in their management, or more capacity for dealing with them? If sound politics demand, above all things, knowledge and capacity, then the sound politics of the habitual drunkards question demand above all things the intervention and experienced judgment of the members of the medical profession. Doctors, it is most true, have played a very small part in the political history of our country in the near and remote past; but the necessities of the State and its population demand that they shall now play a very large part. Not for themselves, but for their country, must doctors assert themselves, and insist upon putting their special knowledge into practice on an imperial scale.

The medical profession, as represented by the British Medical Association, demands the compulsory detention of habitual drunkards, with a view to their cure, and, at any rate, with a view to their being prevented from injuring others. The Home Secretary, if we understand him aright, whilst admitting the necessity for compulsion, desires to see the medical element eliminated from the compulsory process. Therein Mr. Asquith speaks as a lawyer, and as a prejudiced lawyer, not as a statesman. We insist that the medical element is vital to the question. Whilst we would be the last to claim that any habitual drunkard should be deprived of liberty on the sole dictum of any number of doctors, we most firmly assert that no drunkard should be deprived of liberty without the full concurrence of at least one medical man of competent position and authority. A public tribunal, and not private persons, should alone have the power to deprive a man of his liberty, says Mr. Asquith. We quite agree; and we insist that in the case of habitual drunkards that tribunal should consist in a preponderating degree of competent medical men. Only so can justice be reconciled with common sense and the patient's well-being in the case of the habitual drunkard and his friends and the community. For doctors who understand all about the case of the habitual drunkard to be mere certifying witnesses, whilst lawyers, who understand nothing about it, are to be the deciding judges, is one of those absurd anomalies which nothing but centuries of familiarity with absurd anomalies in the law could make us tolerate for a moment. "The tools to him who can handle them" is the rule of right reason, and at this moment more than ever before, of political necessity. The case of the habitual drunkard, if justice is to be done, must in every instance be decided, not merely by law, but by law interpreted and administered by those who are medically capable of a just and complete comprehension of all the facts.

If we desire an illustration of the small value of the mere lawyer outside his own legal speciality, we have but to consider the operation of the Committee of Arbitration which was formed in the City of London not many months ago for the purpose of dealing with commercial disputes. The testimony we hear from all sides is that all classes of commercial disputes are now settled by this committee of plain men of business in a quarter of the time, and at less than a quarter of the cost, which used to be incurred by setting in motion the slow, ponderous, and often profoundly incompetent machinery of the law. If commercial men are more competent to deal with commercial business than members of the law, so likewise, only in a still more pronounced degree, are doctors possessed of a higher measure of competence to deal with the business of physic.

On the general question as to whether habitual drunkards ought to be compulsorily restrained, experienced medical men have long made up their minds, and the State ought to make up its mind. Put into a nutshell, the case stands thus: The habitual drunkard is for practical purposes insane; his insanity has three main aspects; it is injurious to himself; it is injurious to others; and it is in most cases curable by one method, but by one only. Now the State not only has the right, but it is bound, as far as possible, to prevent the habitual drunkard from injuring others. It has also the right, since it has the power—and the prevention of self-injury is a common function of the State—to prevent him injuring himself. When to these considerations is added the third, that the habitual drunkard may be cured of his injurious aberrations by means which involve neither loss nor danger, it is difficult to see how a modern State, which professes to rule only "for the greatest good of the greatest number," can logically avoid the responsibility of curing every habitual drunkard by the one almost certain means which lies within its power. It is admitted that personal liberty is a fundamental right of a citizen of a free State. But it is also affirmed that the power to order deprivation of personal liberty, on adequate cause shown, is a fundamental duty of free governments. What cause can be more adequate, what other cause is ever adduced, for deprivation of liberty than that the deprived person is injurious either to others or to himself? In the case of the habitual drunkard we have every reason for deprivation of personal liberty which is universally allowed, and, in addition, one more, namely, the physical and mental salvation of the drunkard himself. As to the question of process, that is a detail. Let the habitual drunkard's liberty be as carefully safeguarded as the liberties of all other persons by all means; but when that is done, let the insane man be treated as insane, and compulsorily restrained for his own advantage and that of the whole community.

